# Functional apoptosis profiling reveals vulnerabilities in T-cell large granular lymphocytic leukemia

**DOI:** 10.1007/s00277-025-06230-3

**Published:** 2025-02-06

**Authors:** Evgenii Shumilov, Paolo Mazzeo, Marcel Trautmann, Lena Levien, Kerstin Menck, Katharina Richter, Katharina Markus, Lena Ries, Detlef Haase, Elena Oberle, Philipp Berning, Wolfgang Hartmann, Philipp Ströbel, Andrea Kerkhoff, Georg Lenz, Gerald Wulf, Raphael Koch

**Affiliations:** 1 Department of Medicine A, Hematology, Oncology and Pneumology, University Hospital Münster (UKM), Münster, Germany; 2 Department of Hematology and Medical Oncology, University Medical Center Göttingen (UMG), Göttingen, Germany; 3 Department of Hematology and Medical Oncology, INDIGHO laboratory, University Medical Center Göttingen (UMG), Göttingen, Germany; 4 Division of Translational Pathology, Gerhard-Domagk-Institute of Pathology, University Hospital Münster (UKM), Münster, Germany; 5 Department of Pathology, University Medical Center Göttingen (UMG), Göttingen, Germany

**Keywords:** T-cell large granular lymphocytic leukemia (T-LGLL), MCL1, *STAT3*, Apoptosis, BH3 profiling

## Abstract

T-cell large granular lymphocytic leukemia (T-LGLL) is a rare hematologic neoplasm characterized by clonal expansion of CD3 + cytotoxic T lymphocytes and a highly heterogeneous clinical course. Conventional therapy primarily includes immunosuppressive regimen. However, optimal front-line approaches still need to be defined and refractory disease remains a clinical challenge. Thus, we here aimed to explore functional dependencies of T-LGLL as a basis for personalized therapeutic strategies. We performed functional apoptosis profiling and ex vivo drug treatment in a series of 8 clinically and genetically characterized T-LGLL patients from two German University hospitals. Our series of patients underscored the clinical and genetic heterogeneity of the disease. Genetically, only 2 patients harbored a *STAT3* mutation. To identify targetable anti-apoptotic mechanisms, we performed selective functional BH3 profiling on the patients’ CD8 + T-cells harboring the malignant T-LGLL cells versus the same patients’ normal CD4 + T-cells. CD8 + cells in 50% of the patients (4/8) demonstrated a dominant functional dependence on MCL-1 as compared to the same patients’ normal T-cells. Accordingly, CD8 + T-LGLL cells from patients with enhanced MCL1 dependence significantly responded to AZD-5991 ex vivo while no response was observed in the remaining samples lacking enhanced MCL-1 dependence. Across clinically and genetically heterogeneous cases of T-LGLL, functional apoptosis profiling identified patients with CD8 + T-LGLL cells harboring a dominant dependence on MCL-1 as a potential therapeutic target.

**Supplementary Information:**

The online version contains supplementary material available at 10.1007/s00277-025-06230-3.

## Key points

Our study highlights the clinical, genetic and functional heterogeneity of T-LGLL and provides an approach to decipher targetable anti-apoptotic mechanisms. In our series of T-LGLL patients, we provide insight in the heterogeneous functional dependence of T-LGLL cells on MCL-1 and effective induction of apoptosis using AZD-5991 in T-LGLL cells with enhanced MCL-1 dependence.

**Supplementary Information:**

The online version contains supplementary material available at 10.1007/s00277-025-06230-3.

## Introduction

T-cell large granular lymphocytic leukemia (T-LGLL) is a chronic lymphoproliferative disorder characterized by the clonal expansion of CD3 + cytotoxic T lymphocytes [1, 2]. With an incidence of 0.72 per 1,000,000 individuals per year, T-LGLL is considered a rare disease [3]. Median age at diagnosis is typically around 60 years with males and females affected equally [4]. Clinically, T-LGLL offers a variety of manifestations. At diagnosis, T-LGLL presents either asymptomatic with lymphocytosis or symptomatic with constitutional symptoms, cytopenias and possibly recurrent infections. Furthermore, autoimmune phenomena such as rheumatoid arthritis might be associated with T-LGLL. Of note, cytopenia can range from isolated neutropenia to bi- or pancytopenia including pure red cell aplasia (PRCA) and/or rarely thrombocytopenia and result in transfusion dependency.

The indolent course of T-LGLL is the most common form of the disease while aggressive T-LGLL course is documented in distinct cases. Typically, T-LGLL demonstrates a classical leukemic course with presentations in peripheral blood (PB) and bone marrow (BM), while organ manifestations beyond splenomegaly are less frequent [4].

The diagnostic workup of T-LGLL is based on the identification of a persistent T-LGLL peripheral expansion (> 0.5 × 10^9^/L) with surface markers compatible predominantly with an activated T-cell phenotype (CD3+/CD8+/CD57+) and clonal rearrangement of T-cell receptor (TCR) gene [5]. Although diagnostic criteria and clinical features of classical T-LGLL are well established, challenges may arise in cases when T-LGLL expansion remains underrepresented and hardly noticeable in PB and/or BM.

In accordance to the clinical course, on the molecular genetic level T-LGLL also demonstrates a heterogeneous pattern. Particularly, around 40% of T-LGLL cases harbor activating mutations in the signal transducer and activator of the transcription 3 (STAT3) gene while the remaining patients present with wild-type *STAT3* [6, 7]. Activation of STAT3 is considered as an important element in the pathogenesis of T-LGLL and contributes to dysregulation of apoptosis through expression of anti-apoptotic BCL-2 family members [1, 2]. Of these, the anti-apoptotic protein MCL-1 was dominantly suggested as an important pro-survival factor and potential therapeutic target in T-LGLL [8, 9].

For symptomatic patients requiring treatment, immunosuppressive regimen including methotrexate, cyclophosphamide, or cyclosporine are widely used and may induce remissions in some patients [10]. However, effective treatment approaches for refractory T-LGLL patients represent a challenging and unmet medical need that requires further characterization of T-LGLL on the cellular, functional and molecular genetic level.

Aiming to explore the functional dependencies of T-LGLL as a basis for personalized therapeutic strategies, we performed functional apoptosis profiling and ex vivo drug treatment in a series of 8 patients with T-LGLL.

## Materials and methods

### Patients

This study enrolled 8 consecutive T-LGLL patients with available cryopreserved PBMCs, diagnosed and treated between November 2013 and September 2023 in two academic hospitals: University Hospital Göttingen and University Hospital Münster, both Germany. Clinical data were gathered from the medical records electronic patient files, electronic database of the hospitals, or supplemented by additional patient-related documents. All 8 patients signed informed patient consent for local biobanking, research and publication of clinical and research data within this study, in accordance with the Declaration of Helsinki and active protocols approved by the local ethics committees.

### Extraction of DNA

DNA were extracted from PMBC specimens using the AllPrep DNA/ RNA FFPE KIT 50 (Qiagen, Hilden, Germany). Concentrations were determined by fluorometer using Qubit 1X dsDNA HS Assay Kit (Thermo Fisher Scientific, Waltham, Massachusetts, USA).

### Molecular genetics and targeted sequencing

For patients from Göttingen, next-generation sequencing (NGS) using a targeted panel, including up to 54 genes frequently mutated in myeloid neoplasms, or Sanger sequencing was performed as previously described [11]. The identification of T-cell clonality was performed using a PCR-CE–based assay based on the EuroClonality/BIOMED-2 for ABI Fluorescence Detection (Invivoscribe Inc., San Diego, CA) [12, 13].

In addition, all the patients were screened for comprehensive genomic profiling (CGP) adopting the CE-IVD approved OncoDNA OncoDEEP DNA kit (Division of Translational Pathology, Gerhard-Domagk-Institute of Pathology, University Hospital Münster). This technique provided a > 1 MB pan-cancer NGS panel powered by Twist Bioscience hybrid capture enrichment and library preparation technology in combination with OncoDNA’s bioinformatic data analysis and clinical interpretation software suite (OncoKDM and MERCURY). The panel covers 638 cancer-related genes to screen for single-nucleotide variants (SNVs), copy number variants (CNVs), deletions /insertions (indels), loss of heterozygosity (LOH) and facilitates the assessment of key complex biomarkers/ signatures such as homologous recombination deficiency (HRD), tumor mutational burden (TMB), or microsatellite instability (MSI). Sequencing was conducted on the NextSeq 500/550 system (Illumina; Mid Output Kit v2.5 150 Cycles).

### BH3 profiling on primary patient samples

For selective BH3 profiling of CD3+/CD8 + T-cells including the the malignant T-LGLL population versus the same patient’s normal CD3+/CD4 + T-cells, all 8 patients were enrolled in the study. Time points of peripheral blood mononuclear cells (PBMC) collection in the course of the disease are illustrated in Table 1. PBMCs were isolated using ficoll gradient centrifugation according to standard protocols. PBMC samples were stained with anti-human CD3 (dye: BV421. Biolegend, clone HIT3a. cat #300434), anti-human CD4 (dye: PE-Cy5. Becton Dickinson, clone Leu-3a. cat # 566923) and anti-human CD8 (dye: PE. Biolegend, clone SK1. cat# 555367) fluorescently labeled antibodies for flow cytometry. Next, functional BH3 profiling was performed as published previously [14, 15]. Briefly, cells were permeabilized with 0.002% digitonin and treated with either BIM at 0.1 mM and 0.01 mM, BAD, HRK and PUMA at 10 mM, and MS1 and FS1 at 1 mM. Dimethylsulfoxide (DMSO) is an organosulfur compound used as a control and Alamethicin is a peptide-antibiotic that induces pores in the mitochondrial membrane and thus serves as a positive control. The BIM peptide activates the pro-apoptotic effector proteins BAX and BAK and thus reveals a cell’s capability of undergoing apoptosis. BAD antagonizes BCL-2, BCL-xL and BCL-w. HRK specifically antagonizes BCL-xL. MS1 specifically antagonizes MCL1. FS1 specifically antagonizes BFL1. PUMA is a pan-sensitizer (as well as contributing to activating BAX and BAK) and through its BH3 domain neutralizes antiapoptotic Bcl-2 members. Cells were incubated with the peptides for 1 h at 25 °C and subsequently fixed with 4% paraformaldehyde for 10 min. Finally, intracellular cytochrome c was stained with an immunofluorescence-labeled antibody (dye: Alexa fluor. Biolegend, clone 6H2.B4 cat. # 612310) and cells were subjected to flow cytometry to assess cytochrome c release in the patients’ malignant T-LGLL population and CD3+/CD4+ normal T-cells. Relative cytochrome c release in each population was assessed by 1-[(sample-pos.ctrl.)/(neg.ctrl.-pos.ctrl.)].

### Ex vivo treatment

For ex vivo treatment, PBMCs (Table 1) were treated overnight using the MCL-1 specific inhibitor AZD5991 (100nM) and DMSO as a negative control. Cell counts and the viability of cells were assessed by flow cytometry analysis using Annexin V (dye: FITC. Biolegend, cat. #640906). Cells were collected after the treatment, washed, and re-suspended in 100 µl of Annexin V Binding Buffer (Biolegend, cat. #422201), and then stained with 3 µl of Annexin V, together with anti-human CD3, CD4 and CD8 in the dark for 20 min at room temperature. The percentage of relative cell counts was assessed using flow cytometry (BD LSR Fortessa-X20 Analyzer; BD Bioscience).

### Statistics

Statistical analysis was performed using Prism 10.1.2 (GraphPad Software, LCC). Two way ANOVA and Bonferroni post-test were performed both for BH3 profiling and ex vivo treatment. Data are presented as mean ± standard error of the mean (SEM) unless stated otherwise. *p*-values were annotated as follows: * *p* < 0.05, ** *p* < 0.01, *** *p* < 0.001.

## Results

### Clinical and laboratory characteristics of the patients

The clinical and laboratory characteristics of the patients #1–8 are summurized in Table 1. Acknowledging the unusial T-LGLL coure and treatment in patient #1, the details to that are given below while the descprition of cases #2–8 is presented additionaly in Supplemental Material **“Patient #2–8 description”**.

**Fig. 1:**
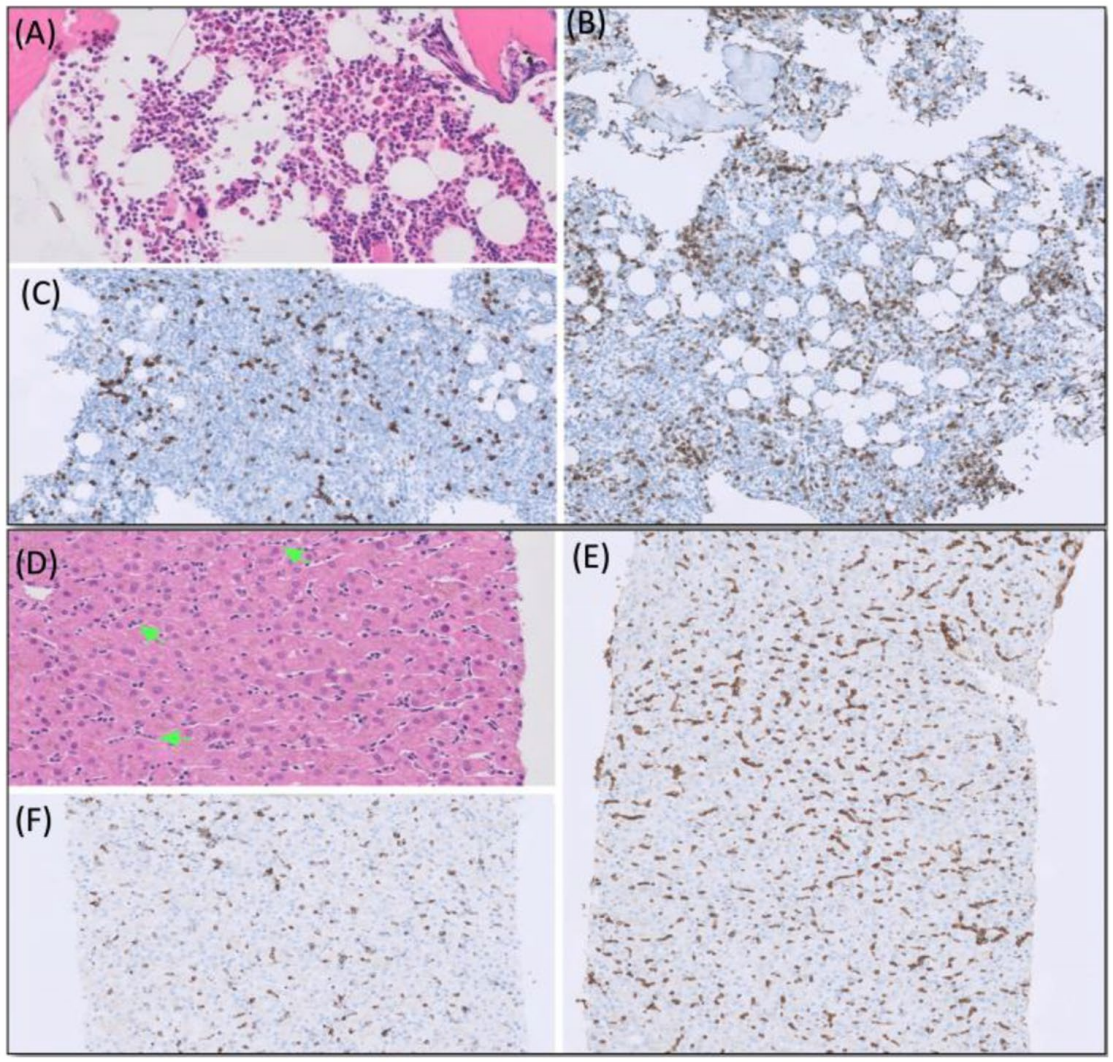
Pathomorphological analysis performed on patient #1. Bone marrow trephine (**A-C**) showing normocellular hematopoiesis on H&E staining. Immunohistochemical CD3 staining (**A**) showed marked diffuse increase of T cells (**B**) with a moderately increased proportion of CD57 positive cells (**C**). Immunoperoxidase on paraffin, initial magnification x200). Liver biopsy (**D-F**) show marked diffuse increase of intrasinusoidal lymphocytes (green arrows) on H&E staining (**D**). On immunohistochemistry, these lymphocytes were CD3 positive T cells (**E**) with a high percentage of CD57 positive cells (**F**). (Immunoperoxidase on paraffin, initial magnification x200)

**Patient #1**, a 28-year-old patient with no previous co-morbidities presented in the emergency department with a transitory ischemic attack triggered by critical hypoxemia due to severe and isolated anemia (Hb 3.1 g/dl) without bleeding sings. Differential blood count revealed normal leukocyte counts with moderate relative neutropenia (22%) and lymphocytosis (72%). Notably, large granular lymphocytes were not detectable by light microscopy. BM aspirate and histology resulted in the diagnosis of a pure red cell aplasia (PRCA) accompanied by an increase of CD3-positive T-cells (Fig. 1A-C). Chromosome banding, fluorescence in situ hybridization (FISH), and initial next generation sequencing (NGS) panel (Qiagen, 49 genes frequently altered in myeloid malignancies) performed on BM aspirate were inconspicuous. Notably, fluorescence-activated cell sorting (FACS) of PB revealed a small aberrant T-cell population (1432/µl; CD3+/CD8+/CD7-/CD56-/CD57+/TCRαβ) with an inverted CD4+/CD8+ ratio of 0.3. The analysis of T-cell receptor (TCR) gene rearrangement by PCR in PB confirmed the clonality of T-lymphocytes with rearrangement of the ɣ-chain. Finally, CT scan had showed considerable hepatosplenomegaly (HSM) resulting in liver biopsy. The pathomorphological analysis of liver sample revealed T-cell infiltrates (Fig. 1D-F) with above mentioned TCR rearrangements and confirmed the diagnosis of T-LGLL. Furthermore, extended NGS identified a *STAT3 c.1981G > T* (NM_139276.3:c.1981G > T; NP_644805.1:p.D661Y; 27.7% VAF) mutation. Of note, transaminase levels were normal at diagnosis.

The patient received front-line treatment with prednisone and methotrexate (MTX) (15 mg s.c. weekly) according to the LGL1 protocol (NCT00278265). During the following 6 months on treatment, the patient remained refractory with an ongoing need for transfusions and a persistent T-LGLL cell population in PB confirmed by FACS. Due to this refractory clinical course, the patient underwent salvage allogeneic stem cell transplantation (allo-SCT) from a mismatched unrelated donor after myeloablative conditioning with FBC12-ATG regimen: fludarabine 125mg/m^2^, busulfan 9,6 mg/kg body weight, cyclophosphamide 120 mg/kg body weight, antithymocyte globulin 60 mg/kg body weight. At the last follow-up 22 months after allo-SCT, the patient was alive with 100% donor-chimerism and complete remission of T-LGLL. Unfortunately, the post-allo-SCT course was complicated by chronic graft versus host disease (cGvHD).

**Table 1:** Overview on 8 patients of the study

Patient №	Age/Sex	Clinical and laboratory findings at diagnosis of T-LGLL	Organs involved	Histopathology, genetic findings	Treatment / clinical course	PMBC collection
**Patient #1**	28 yrs, male	**Cl**: severe anemia with neurologic symptomatic **PB**: severe anemia, neutropenia, no lymphocytosis FACS (PB): suspicious T-cell population (CD3+/CD7-/CD8+/CD57+) **BM**: PRCA; NGS and cytogenetics inconspicuous TCR analysis (PB): monoclonal T-cell population	L, Spl, BM	liver biopsy: T-LGL infiltrates, mutated *STAT3* (p.D661Y)	1st line: MTX/cortisone with no response; 2nd line: salvage allo-SCT last FU (22 mo): alive in CR after salvage allo-SCT	PD under MTX / cortisone therapy
**Patient #2**	54 yrs, female	**Cl**: history of sero-negative rheumatoid arthritis. Weight loss, lymphadenopathy and incidental finding of thrombopenia and leukopenia. **PB**: thrombopenia, leukopenia with lymphocytosis and severe neutropenia **BM**: 70% T-cell infiltrate FACS (PB and BM): CD3+/CD4-/CD8+/TCRα/β + with decreased expression of CD5, CD2 and CD7. TCR analysis: biclonal ɣ-chain rearrangement	LN, Spl, BM	BM biopsy: T-LGL infiltrate with 70% BM involvement, mutated *STAT3* (p.Y640F)	1st line: cyclophosphamide	Prior to cyclophos-phamide
**Patient #3**	51 yrs, female	**Cl**: Incidental finding of severe neutropenia with lymphocytosis **PB**: isolated severe neutropenia **BM**: T-cell infiltrates FACS (PB): clonal T-cell population CD3+/CD4-/CD8+/CD16-/CD56-/TCR ɣ/ð+ TCR analysis (BM): rearrangement of β- and ɣ-chains (T-cell biclonal population)	LN, Spl, BM	BM biopsy: T-cell infiltrates, wild-type *STAT3/STAT5b*	1st line: watch & wait Last FU (8 mo): death due to fulminant pneumonia without preceding T-LGL therapy	Prior to any therapy
**Patient #4**	52 yrs, male	**Cl**: history of renal transplant with ongoing triple immunosuppression. Progressive anemia, lymphocytosis and splenomegaly. **PB**: severe anemia, T-cell lymphocytosis **BM**: 20% T-cell population FACS: CD3+/CD4-/CD5+/CD7+/ CD8+/CD16+/CD56- TCR analysis: rearrangement of β- and ɣ-chains (T-cell biclonal population)	Spl, BM	BM biopsy: T-cell population up to 20%, wild-type *STAT3/STAT5b* Mutated *TET2* (p. Y370*)	1st line: watch & wait Last FU (5 mo): alive, stable course, anemia requires no blood transfusions	Prior to any therapy
**Patient #5**	68 yrs, female	**Cl**: previous history of rheumatoid arthritis **PB**: bicytopenia **BM**: T-cell infiltrates FACS/IHC (BM): CD3+/CD4-/CD5-/CD7+/CD8weak/CD56-/CD57-/TCR ɣ/ð+ TCR analysis (BM): rearrangement of ɣ-chain (T-cell monoclonal population)	Spl, BM	BM biopsy: T-cell infiltrates, wild-type *STAT3/STAT5b*	1st line: MTX 2nd line: Cy/CyA 3nd line: G-CSF Last FU (18 mo): alive, mild pancytopenia	Prior to any therapy
**Patient #6**	74 yrs, male	**Cl**: severe recurrent infections **PB**: mild anemia only **BM**: T-cell infiltrates IHC/FACS (BM): suspicious T-cell population (CD3+/CD4-/CD8+/CD56+/CD57+) TCR analysis (BM): rearrangement of β- and ɣ-chains (T-cell biclonal population)	BM	BM biopsy: T-cell infiltrates, wild-type *STAT3/STAT5b*	1st line: MTX followed by MTX/prednisone Last FU (8 mo): alive, reduction of infections, normal blood count	Under ongoing MTX/prednisone therapy
**Patient #7**	53 yrs, male	**Cl**: recurrent infections, constitutional symptoms **PB**: isolated severe neutropenia **BM**: T-cell infiltrates IHC/FACS (BM): suspicious T-cell population (CD3+/CD4-/CD8+/CD56+/CD57+) TCR analysis (BM): rearrangement of β- and ɣ-chains (T-cell biclonal population)	BM	BM biopsy: T-cell infiltrates, wild-type *STAT3/STAT5b*	1st line: watch & wait, social distance Last FU (136 mo): alive, stable course, still no therapy indication	Prior to any therapy
**Patient #8**	56 yrs, female	**Cl**: constitutional symptoms with previous history of schwannomatosis **PB**: normal blood count **BM**: up to 15% T-cell population FACS/IHC (PB/BM): suspicious T-cell population CD3+/CD4-/CD5+/CD7+/CD8+/CD56-/CD57+ TCR analysis (BM): clonal TCR rearrangements in β- and ɣ-chains	BM	BM biopsy: T-cell population (15%), wild-type *STAT3/STAT5b*	1st line: MTX with no improvement of constitutional symptoms Last FU (5 mo): alive, no therapy	2 weeks after start of MTX therapy

T-LGLL: T-cell large granular lymphocytic leukemia; yrs: years; Cl: clinical presentation; PB: peripheral blood; FACS: fluorescence-activated cell sorting, CD: cluster of differentiation, BM: bone marrow, PRCA: pure red cell aplasia, NGS: next generation sequencing, TCR: T-cell receptor; CT: computed tomography; L: liver; LN: lymph node; Spl: spleen; MTX: methotrexate, Cy: cyclophosphamide; FU: follow-up; mo: month; SD: stable disease; PD, progressive disease; CyA: cyclosporin A; NA, not available

### Comprehensive genomic profiling of mononuclear cells from T-LGLL patients

The results of targeting sequencing of mononuclear cells from T-LGLL patients are presented in Table 2. Of six patients, two patients (#1, #2) presented with a *STAT3* mutation: p.D661Y (27.7% VAF) and p.Y640F (24%VAF), respectively. Particularly, we did not identify mutations or copy number alterations in *MCL1* among all patients. Thus, two of four patients with enhanced MCL1 dependency according to BH3 profiling (#1–4) harbored a somatic *STAT3* mutation. Beyond *STAT3*, our targeted NGS panel only revealed mutations in *TPMT* in two cases (#3–4) and *TET2* in one case as potentially pathogenic variants. All patients were tested negative for HRD, TMB low and MSI as summarized in Table 2.

**Table 2:** Targeting sequencing of mononuclear cells from T-LGLL patients

Patient №	STAT3/STAT5 mutation status	Other pathogenic/likely pathogenic variants	HRD	TMB	MSI
#1	*STAT3* c.1981G > T, p.D661Y, VAF 26%	no	neg. (4.0)	low (4.45 Mut/Mb)	stable
#2	*STAT3* c.1919 A > T, p.Y640F, VAF 24%	no	neg. (12.0)	low (4.45 Mut/Mb)	stable
#3	wild-type	*TPMT*, VAF 100%	neg. (9.0)	low (5.01 Mut/Mb)	stable
#4	wild-type	*TET2*, VAF 38%	neg. (8.0)	low (1.11 Mut/Mb)	stable
#5	wild-type	*TPMT*, VAF 100%	neg. (4.0)	low (2.22 Mut/Mb)	stable
#6	wild-type	no	neg. (10.0)	low (2.23 Mut/Mb)	stable
#7	wild-type	no	neg. (10.0)	low (2.78 Mut/Mb)	stable
#8	wild-type	no	neg. (9.0)	low (1.11 Mut/Mb)	stable

T-LGLL, T-cell large granular lymphocytic leukemia; VAF: variant allele frequency. HRD: homologous recombination deficiency, TMB: tumor mutational burden, MSI: microsatellite instability

### Characterization of mitochondrial apoptosis in T-LGLL cells according to functional BH3 profiling

**Fig. 2:**
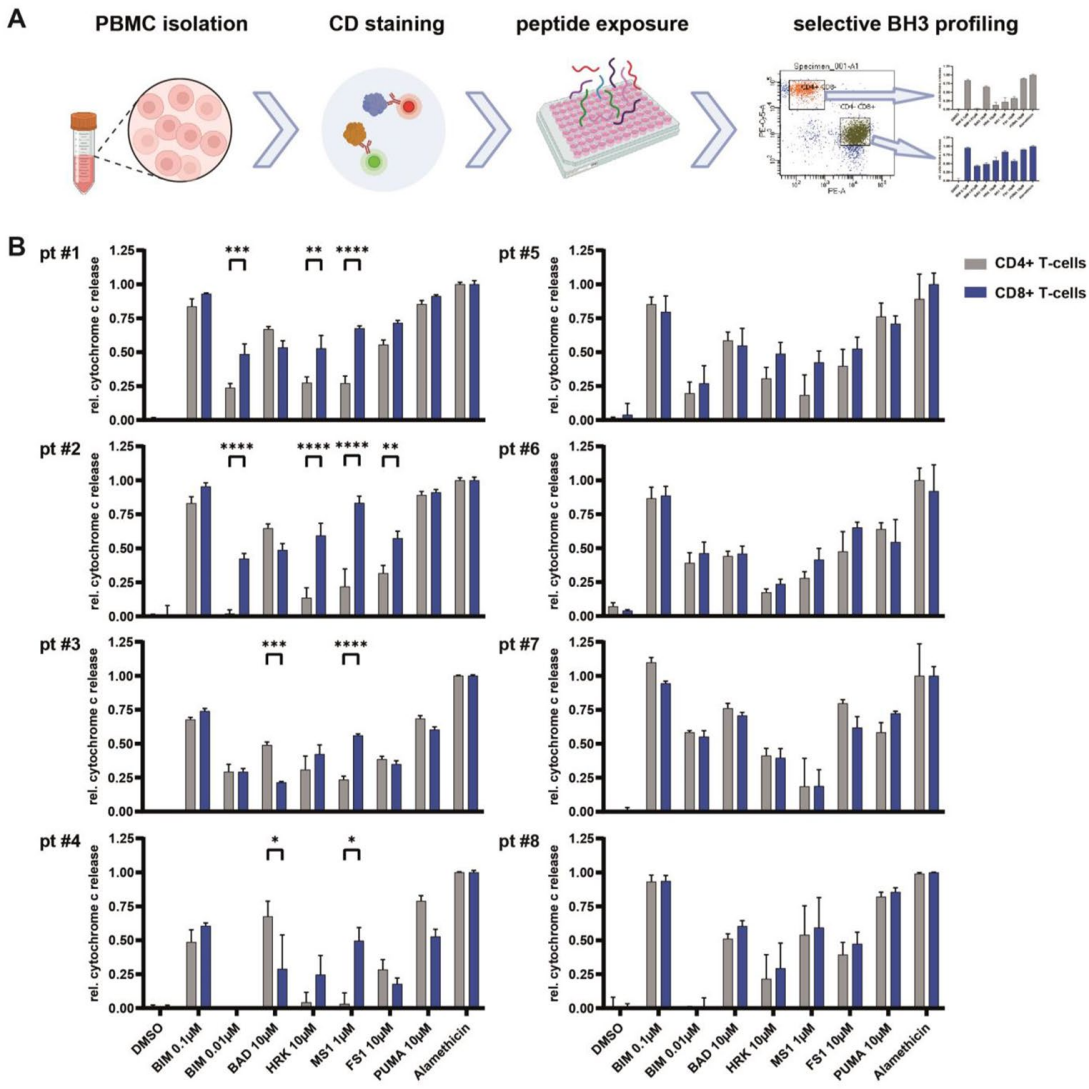
Functional apoptosis profiling of T-LGLL versus normal T-cells in patients with T-LGLL. (**A**) Workflow and (**B**) results of selective, functional BH3, demonstrating heterogeneous responses to pro-apoptotic activators and sensitizers revealing diverse apoptotic priming and dependence on key anti-apoptotic proteins (BCL2, BCL-xL, BCL-w, BFL1, and MCL1) within CD8+ T-cells (harboring T-LGLL clones, in blue) as compared to the same patients’ CD4+ T-cells (in grey). The first four patients (#1–4) showed a dominant response to the pro-apoptotic sensitizer MS1 indicating functional dependence on MCL-1. In contrast, the CD8+ T-cells from the other three patients (#5–8) lacked significant differences in apoptotic dependencies

The results of apoptotic priming and functional significance of anti-apoptotic proteins in T-*LGLL* samples of the patients are presented in Fig. 2. In 4 patients (#1–4), BH3 profiling identified distinct, but heterogeneous responses to the pro-apoptotic activator BIM and the pro-apoptotic sensitizers BAD, HRK, FS1 and PUMA, indicating heterogeneous apoptotic priming and heterogeneous dependence on the anti-apoptotic proteins BCL2, BCL-xL and BCL-w and BFL-1, in CD8 + cells harboring the malignant T-LGLL clone as compared to the same patients’ CD+ T-cells. Significantly, BH3 profiling demonstrated a predominant response to the pro-apoptotic sensitizer MS1 in T-LGLL cases #1–4 (Fig. 2), suggesting an enhanced functional dependence on MCL1 of CD8+ T-cells including the malignant T-LGLL clone. Additionally, we observed a notable difference between the two examined cell populations concerning responses to BIM, BAD, HRK and FS1 in individual cases: CD8+ cells of cases #1 and #2 not only showed increased susceptibility to MS1 but also to HRK and BIM, indicating enhanced functional dependence on BCL-xL and increased activation of pro-apoptotic activators through BIM, which is also referred to as apoptotic priming. Furthermore, CD8+ cells of patients #3 and #4 showed decreased susceptibility to BAD without significant changes in response to HRK. This finding strongly suggests a decreased functional dependence on BCL-2 in CD8+ T-cells versus the same patients CD4+ T-cells. Importantly, CD8+ versus CD4+ T-cells from the remaining four patients (#5–8) did not exhibit statistically significant differences in apoptotic priming, including functional dependence on MCL1.

DMSO and Alamethicin served as negative and positive controls, respectively. The BIM peptide activates the pro-apoptotic effector proteins BAX and BAK and thus reveals a cell’s capability of undergoing apoptosis, also referred to as apoptotic priming. BAD antagonizes BCL-2, BCL-xL and BCL-w. HRK specifically antagonizes BCL-xL. MS1 specifically antagonizes MCL-1. FS1 specifically antagonizes BFL1. PUMA is a pan-sensitizer.

Bar graphs indicate the mean of a technical triplicate. Error bars indicate the standard error of mean. Two way ANOVA and Bonferroni post-test were performed. * *p* < 0.05, ** *p* < 0.01, *** *p* < 0.001, **** *p* < 0.0001.

### Ex vivo treatment with the MCL-1 inhibitor AZD-5991 in T-LGLL samples

**Fig. 3:**
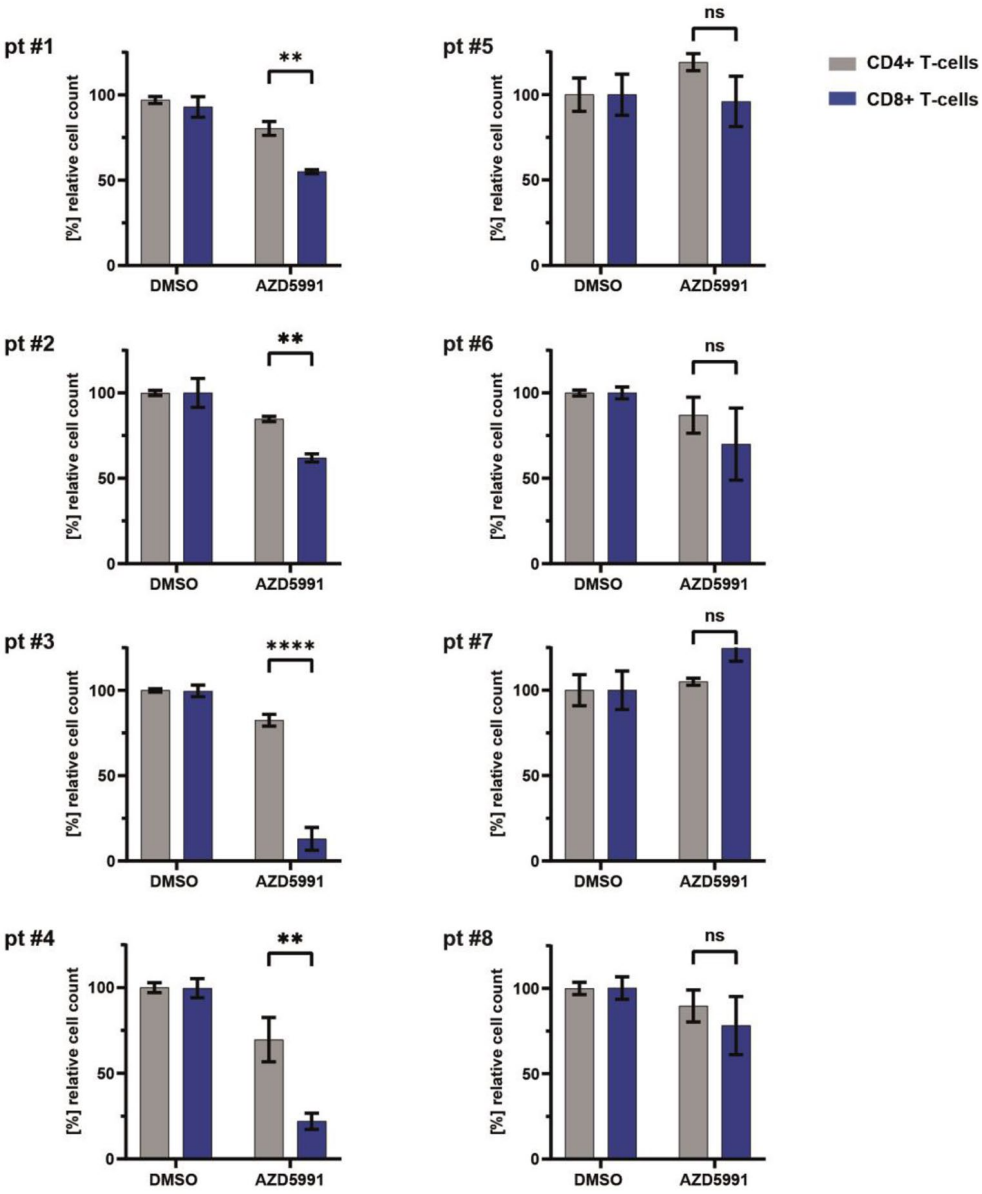
Efficacy of ex-vitro treatment with the MCL-1 inhibitor AZD-5991 in T-LGLL samples. Patients’ PBMCs were treated with DMSO and 100nM AZD5991 for 24 h. Viable CD8+ T-cells (harboring the malignant T-LGLL clone) and CD4+ T-cells were quantified by flow cytometry. CD8+ T-cells of patients #1–4 showed a significant reduction as compared to the same patients‘ CD4+ T-cells, while CD8+ T-cells from patients #5–8 did not show a significantly reduced viability as compared to CD4+ T-cells. Bar graphs indicate the mean of a technical triplicate. Error bars indicate the standard error of mean. Two way ANOVA and Bonferroni post-test were performed. * *p* < 0.05, ** *p* < 0.01, *** *p* < 0.001, **** *p* < 0.0001

For all patients included in our cohort, sufficient numbers of cryopreserved viably PBMCs were available for ex vivo drug testing. We treated the patients’ PBMCs ex vivo with the MCL-1 specific inhibitor AZD-5991. Cytotoxic responses to AZD-5991 of the patients’ CD8+ T-cells harboring the malignant T-LGLL clone versus the same patients’ CD4+ T-cells were determined by flow-cytometry based viable cell counts and are presented in Fig. 3. Strikingly, CD8+ T-cells from all four patients with enhanced MCL-1 dependency described above demonstrated a significantly enhanced response to AZD-5991 (patient #1–2 and ‘4: *p*-value ≤ 0.01; patient #3: *p*-value: ≤ 0.0001) in comparison to the same patient’s CD4+ T-cells. Notably, viable cell counts of CD8+ T-cells from the remaining patients (#5–8) without enhanced MCL-1 dependency by BH3 profiling were not significantly more affected by AZD-5991 than these patients’ CD4+ T-cells (Fig. 3A).

## Discussion

We here comprehensively characterized a series of eight T-LGLL patients on the clinical, genetic and functional level aiming to improve the knowledge about this entity and provide a strategy for rational targeting of anti-apoptotic mechanisms.

Clinically, we observed a very heterogeneous pattern varying from asymptomatic course to severe cytopenia presented by isolated/combined neutropenia, thrombocytopenia and anemia including PRCA, recurrent infections, constitutional symptoms as well as liver involvement. As illustrated in case #1 from our patient cohort, T-LGLL cells can escape the classical morphological diagnosis following thorough examination of PB and/or BM and disease can present by organ manifestations. Although being a rare event, the latter may vary broadly and affect such sites as liver, intestine, lungs and even chorea according to the literature (Supplemental Table S1) [16–20]. Thus, T-LGLL can represent a challenging diagnosis requiring a thorough diagnostic workup, often as part of a multidisciplinary approach. Finally, the screening for organ manifestations should be initiated in situations suspicious but not fully verifiable for T-LGLL in PB and/or BM.

Immunosuppressive drugs such as MTX, cyclophosphamide, cyclosporine A, and prednisone represent widely used treatment options for patients with T-LGLL [10]. So far, allo-SCT represents a rescue option for refractory T-LGLL cases. Although the data are still scarce, promising results for allo-SCT were reported by single reports including our case [21–23]. Considering transplantation related mortality and morbidity, there is a need for novel therapeutic strategies in refractory cases of T-LGLL. Our findings as well as previous studies point out that MCL-1 can be an attractive target in treatment of T-LGLL.

Meanwhile novel compounds targeting MCL-1 in hematologic malignancies are entering clinical trials [24]. However, strategies to identify patients that might benefit from MCL-1 directed therapies are still needed.

To functionally dissect the apoptosis-machinery in T-LGLL, we performed selective BH3 profiling [14, 15] of CD8+ T-cells harboring the malignant T-LGLL clones versus the same patient’s CD4+ T-cells. In accordance with the aforementioned clinical heterogeneity, BH3 apoptosis profiling also revealed heterogeneous apoptotic priming and anti-apoptotic dependencies across the included cases of T-LGLL.

Nevertheless, our approach, which relies on flow cytometry technology to ensure the purity of malignant T-LGLL cells within the CD8+ cell population, may introduce a potential bias in the results.

Notably, half of the patients (4/8) exhibited enhanced dependency on the anti-apoptotic protein MCL-1 of the CD8+ T-cells harboring the malignant T-LGLL clone in comparison to the patients’ CD4+ T-cells, while the other half did not. Previous studies on T-LGLL have generally indicated uniform upregulation of the pro-survival protein MCL-1 in T-LGLL cells, promoting anti-apoptotic mechanisms and proliferation [8, 9]. Notably, we did not detect any MCL-1 amplification in our cohort which could potentially correlate with MCL-1 dependency, thereby promoting, for instance, cancer survival and drug resistance [25, 26]. Furthermore, assessing the functional and therapeutic relevance of MCL1 based solely on gene expression levels is challenging, as additional factors such as protein stability and degradation strongly influence its function [27]. In contrast, BH3 profiling directly assesses the functional relevance of MCL1 as a potential therapeutic target in cancer cells [15, 28]. In our study we demonstrate heterogeneous functional dependence on MCL-1 in cases of CD8+ T-LGLL. Along this line, inhibition of MCL-1 ex vivo resulted in markedly reduced T-LGLL clone viability only in cases with enhanced MCL-1 dependence by BH3-profiling. In addition, previous studies demonstrated that mutated *STAT3* upregulates MCL-1 in T-LGLL [29]. In our study, MCL-1 dependency was documented independently of the *STAT3* mutation status and across the clinical heterogeneity of the cases.

Beyond the results of our study, which specifically focussed on tumorcell-autonomous mechanisms, recent research also highlights the relevance of the dysregulated underlying immune system and the potential of therapies targeting the entire immune repertoire, rather than solely the malignant T-LGLL clone [30]. In line with this, it is possible that drugs like cyclophosphamide achieve their clinical activity by attenuating the entire immune system. The role of anti-apoptotic proteins in this context currently remains elusive. Interestingly though, single-cell analyses of Huuhtanen et al. revealed MCL1 as a differentially expressed gene in T-LGLL clusters [30]. Together with the established functional role of MCL-1 in the pathogenesis of T-LGLL as a pro-survival factor of the malignant clone [2, 9, 31], these results should prompt further studies to systematically evaluate therapeutic strategies targeting both the malignant T-LGLL clone and the broader immune system.

## Conclusions

Taken together, T-LGLL represents a highly heterogeneous hematologic malignancy on clinical, functional and molecular-genetic level. The presence of MCL-1 dependence in CD8+ T-LGLL cases is variable and not compulsory associated with mutated *STAT3*. In our heterogeneous series of T-LGLL cases, functional BH3 profiling identified cases of CD8+ T-LGLL with enhanced MCL1 dependence and sensitivity to the MCL1 inhibitor AZD5991 as compared to the same patient’s CD4+ T-cells. These results exemplify a strategy to identify potentially targetable anti-apoptotic mechanisms in T-LGLL. Still, additional studies are needed to assess the clinical benefit of apoptosis-directed therapies in T-LGLL and to determine how such strategies can be integrated into therapeutic concepts targeting both the malignant T-LGLL clone and the broader dysregulated immune system.

## Electronic supplementary material

Below is the link to the electronic supplementary material.

Supplementary Material 1

## Data Availability

No datasets were generated or analysed during the current study.

## References

[1] Marchand T, Lamy T, Loughran TP (2024) A modern view of LGL Leukemia. Blood 144. 10.1182/BLOOD.2023021790 10.1182/blood.2023021790 38848524

[2] Lamy T, Moignet A, Loughran TPLGL, Leukemia (2017) From pathogenesis to treatment. Blood 129:1082–1094. 10.1182/BLOOD-2016-08-692590 10.1182/blood-2016-08-692590 28115367

[3] Dinmohamed AG, Brink M, Visser O, Jongen-Lavrencic M (2016) Population-based analyses among 184 patients diagnosed with large granular lymphocyte leukemia in the Netherlands between 2001 and 2013. Leukemia 30:1449–1451. 10.1038/LEU.2016.68 27055870 10.1038/leu.2016.68

[4] Oshimi K (2017) Clinical features, Pathogenesis, and treatment of large Granular Lymphocyte Leukemias. Intern Med 56:1759–1769. 10.2169/INTERNALMEDICINE.56.8881 28717070 10.2169/internalmedicine.56.8881 PMC5548667

[5] Bareau B, Rey J, Hamidou M, Donadieu J, Morcet J, Reman O, Schleinitz N, Tournilhac O, Roussel M, Fest T et al (2010) Analysis of a French cohort of patients with large granular lymphocyte leukemia: a report on 229 cases. Haematologica 95:1534–1541. 10.3324/HAEMATOL.2009.018481 20378561 10.3324/haematol.2009.018481 PMC2930955

[6] Muñoz-García N, Jara-Acevedo M, Caldas C, Bárcena P, López A, Puig N, Alcoceba M, Fernández P, Villamor N, Flores-Montero JA et al (2020) STAT3 and STAT5B mutations in T/NK-Cell Chronic Lymphoproliferative disorders of large granular lymphocytes (LGL): Association with Disease features. Cancers (Basel) 12:1–20. 10.3390/CANCERS12123508 10.3390/cancers12123508 PMC7760806 33255665

[7] Koskela HLM, Eldfors S, Ellonen P, van Adrichem AJ, Kuusanmäki H, Andersson EI, Lagström S, Clemente MJ, Olson T, Jalkanen SE et al (2012) Somatic STAT3 mutations in large granular lymphocytic leukemia. N Engl J Med 366:1905–1913. 10.1056/NEJMOA1114885 22591296 10.1056/NEJMoa1114885 PMC3693860

[8] LeBlanc FR, Yang J, Heller JH, Dighe S, Gru AA, Dunlap-Brown M, Wang H-G, Feith DJ, Loughran TP (2017) Therapeutic efficacy of Mcl-1 antagonism in large granular lymphocyte leukemia. Blood 130. 10.1182/blood.V130.Suppl_1.1217.1217

[9] LeBlanc FR, Pearson JM, Tan SF, Cheon HJ, Xing JC, Dunton W, Feith DJ, Loughran TP (2020) Sphingosine Kinase-2 is overexpressed in large granular lymphocyte leukaemia and promotes survival through Mcl-1. Br J Haematol 190:405–417. 10.1111/BJH.16530 32124438 10.1111/bjh.16530 PMC7415522

[10] Cheon HJ, Dziewulska KH, Moosic KB, Olson KC, Gru AA, Feith DJ, Loughran TP (2020) Advances in the diagnosis and treatment of large granular lymphocytic leukemia. Curr Hematol Malig Rep 15:103–112. 10.1007/S11899-020-00565-6 32062772 10.1007/s11899-020-00565-6 PMC7234906

[11] Martin R, Acha P, Ganster C, Palomo L, Dierks S, Fuster-Tormo F, Mallo M, Ademà V, Gómez-Marzo P, De Haro N et al (2018) Targeted deep sequencing of CD34 + cells from Peripheral Blood can reproduce bone marrow Molecular Profile in Myelodysplastic syndromes. Am J Hematol 93:E152–E154. 10.1002/AJH.25089 29575088 10.1002/ajh.25089 PMC6001632

[12] Sandberg Y, Verhaaf B, van Gastel-Mol EJ, Wolvers-Tettero ILM, de Vos J, MacLeod RAF, Noordzij JG, Dik WA, van Dongen JJM, Langerak AW (2007) Human T-Cell lines with Well-defined T-Cell receptor gene rearrangements as controls for the BIOMED-2 Multiplex polymerase chain reaction tubes. Leukemia 21:230–237. 10.1038/SJ.LEU.2404486 17170727 10.1038/sj.leu.2404486

[13] Patel KP, Pan Q, Wang Y, Maitta RW, Du J, Xue X, Lin J, Ratech H (2010) Comparison of BIOMED-2 versus Laboratory-developed polymerase chain reaction assays for detecting T-Cell receptor-Gamma Gene rearrangements. J Mol Diagn 12:226–237. 10.2353/JMOLDX.2010.090042 20181819 10.2353/jmoldx.2010.090042 PMC2871730

[14] Ryan J, Montero J, Rocco J, Letai A (2016) IBH3: simple, fixable BH3 profiling to determine apoptotic priming in primary tissue by Flow Cytometry. Biol Chem 397:671–678. 10.1515/HSZ-2016-0107 26910743 10.1515/hsz-2016-0107

[15] Koch R, Christie AL, Crombie JL, Palmer AC, Plana D, Shigemori K, Morrow SN, Van Scoyk A, Wu W, Brem EA et al (2019) Biomarker-driven strategy for MCL1 inhibition in T-Cell lymphomas. Blood 133:566–575. 10.1182/BLOOD-2018-07-865527 30498064 10.1182/blood-2018-07-865527 PMC6367646

[16] de Mel S, Wong B, Gole L, Ng SB, Koay E, Siong CNW, Seet JE, Wee A, Chng WJ, Tan LK (2015) A rare variant of aggressive T-Cell large granular lymphocyte leukemia Associated with hepatic fibrosis and trisomy 8: a Case Report and Literature Review. J Hematol 4:214–218. 10.14740/JH223W

[17] Li R, Gong P, Gao Z (2016) Diffused pulmonary infiltrates with T-Cell large granular lymphocytic leukemia. Chest 149. 10.1016/j.chest.2016.02.477

[18] Malamut G, Meresse B, Verkarre V, Kaltenbach S, Montcuquet N, Van Duong JP, Callens C, Lenglet J, Rahmi G, Samaha E et al (2012) Large granular lymphocytic leukemia: a treatable form of refractory celiac disease. Gastroenterology 143. 10.1053/J.GASTRO.2012.08.028 10.1053/j.gastro.2012.08.028 22922421

[19] Sarny S, Beham-Schmid C, El-Shabrawi Y (2020) Choroidal Infiltration as First Clinical Manifestation of T-Cell large granular lymphocyte (T-LGL) leukemia. Ocul Immunol Inflamm 28:1133–1135. 10.1080/09273948.2019.1645186 31577464 10.1080/09273948.2019.1645186

[20] Lamy T, Bauer FA, Liu JH, Li YX, Pillemer E, Shahidi H, Gregory SA, Zambello R, Marcolongo R, Semenzato G et al (2000) Clinicopathological features of aggressive large Granular Lymphocyte Leukaemia Resemble Fas ligand transgenic mice. Br J Haematol 108:717–723. 10.1046/J.1365-2141.2000.01934.X 10792274 10.1046/j.1365-2141.2000.01934.x

[21] Carey E, Ward N, Abdul-Hay M (2022) Large granular lymphocytic leukemia cured by allogeneic stem cell transplant: a Case Report. J Med Case Rep 16. 10.1186/S13256-022-03447-Y 10.1186/s13256-022-03447-y PMC9175501 35672859

[22] Marchand T, Lamy T, Finel H, Arcese W, Choquet S, Finke J, Huynh A, Irrera G, Karakasis D, Konopacki J et al (2016) Hematopoietic stem cell transplantation for T-Cell large granular lymphocyte leukemia: a retrospective study of the European Society for Blood and marrow transplantation. Leukemia 30:1201–1204. 10.1038/LEU.2015.256 26460210 10.1038/leu.2015.256

[23] Donato ML, Goldberg SL, Pecora AL, Rowley SD (2007) Treatment of T cell large granular lymphocyte leukemia with reduced-intensity allogeneic stem cell transplantation. Blood 110:4931–4931. 10.1182/BLOOD.V110.11.4931.4931

[24] Wang H, Guo M, Wei H, Chen Y (2021) Targeting MCL-1 in Cancer: current status and perspectives. J Hematol Oncol 14. 10.1186/S13045-021-01079-1 10.1186/s13045-021-01079-1 PMC8061042 33883020

[25] Hird AW, Tron AE (2019) Recent advances in the development of Mcl-1 inhibitors for Cancer Therapy. Pharmacol Ther 198:59–67. 10.1016/J.PHARMTHERA.2019.02.007 30790641 10.1016/j.pharmthera.2019.02.007

[26] Shahar N, Larisch S (2020) Inhibiting the inhibitors: targeting anti-apoptotic proteins in Cancer and Therapy Resistance. Drug Resist Updat 52. 10.1016/J.DRUP.2020.100712 10.1016/j.drup.2020.100712 32599435

[27] Sancho M, Leiva D, Lucendo E, Orzáez M (2022) Understanding MCL1: from Cellular function and regulation to pharmacological inhibition. FEBS J 289:6209–6234. 10.1111/FEBS.16136 34310025 10.1111/febs.16136 PMC9787394

[28] Ryan J, Letai A (2013) BH3 profiling in whole cells by fluorimeter or FACS. Methods 61:156–164. 10.1016/J.YMETH.2013.04.006 23607990 10.1016/j.ymeth.2013.04.006 PMC3686919

[29] Epling-Burnette PK, Liu JH, Catlett-Falcone R, Turkson J, Oshiro M, Kothapalli R, Li Y, Wang JM, Yang-Yen HF, Karras J et al (2001) Inhibition of STAT3 signaling leads to apoptosis of Leukemic large granular lymphocytes and decreased Mcl-1 expression. J Clin Invest 107:351–361. 10.1172/JCI9940 11160159 10.1172/JCI9940 PMC199188

[30] Huuhtanen J, Bhattacharya D, Lönnberg T, Kankainen M, Kerr C, Theodoropoulos J, Rajala H, Gurnari C, Kasanen T, Braun T et al (2022) Single-Cell Characterization of Leukemic and Non-Leukemic Immune Repertoires in CD8 + T-Cell Large Granular Lymphocytic Leukemia. *Nature Communications 2022 13:1 13*, 1–16. 10.1038/s41467-022-29173-z 10.1038/s41467-022-29173-z PMC9001660 35411050

[31] Zhang R, Shah MV, Yang J, Nyland SB, Liu X, Yun JK, Albert R, Loughran TP (2008) Network Model of Survival Signaling in large granular lymphocyte leukemia. Proc Natl Acad Sci U S A 105:16308–16313. 10.1073/PNAS.0806447105 18852469 10.1073/pnas.0806447105 PMC2571012

