# Supplementary material for: Functional apoptosis profiling reveals vulnerabilities in T-cell large granular lymphocytic leukemia
Source: Ann Hematol. 2025 Feb 6;104(1):581–91. doi: 10.1007/s00277-025-06230-3 (PMC11868225; doi:10.1007/s00277-025-06230-3)
Supplement: Supplementary file 1 — Supplementary Material 1 [file 277_2025_6230_MOESM1_ESM.docx]

**Supplemental Table S1. Overview on 9 patients diagnosed with uncommon T-LGL organ manifestations reported in the literature**. T-LGL: T-cell large granular lymphocytic leukemia; yrs: years; PB: peripheral blood; FACS: fluorescence-activated cell sorting, CD: cluster of differentiation, BM: bone marrow, PRCA: pure red cell aplasia, NGS: next generation sequencing, TCR: T-cell receptor; CT: computed tomography; HSM: hepatosplenomegaly; MTX: methotrexate, Cy: cyclophosphamide; FU: follow-up; mo: month; SD: stable disease, CyA: cyclosporin A; CHOP: cyclophosphamide; doxorubicin; vincristin; prednisone; NA, not available.

* two previous therapies mentioned but not specified

| **Sarny et al.(19),**  **2019** | 55 yrs, female | photophobia followed by lymphocytosis 4 months later  PB: isolated lymphocytosis 15.000/µl, no cytopenia  BM: T-LGL infiltrates | eye exam: choroidal lesions in both  eyes | biopsy of choroid:  T-LGL infiltrates | 1^st^ line: Cy followed by addition of MTX and intravitreal dexamethasone;  last FU (8 mo): alive; gain of vision, no other systemic symptoms or cytopenias |
| --- | --- | --- | --- | --- | --- |
| **Li et al.(17),**  **2016** | 52 yrs, female | cough and dyspnea for one and a half years before admission  PB: cytopenia (non-specified)  PB/BM on admission: lymphocytosis with microscopy and FACS in consistence with T-LGL | CT: bilateral diffused pulmonary infiltrates | lung biopsy: T-LGL infiltrates | 1^st^ line: MTX/cortison;    last FU (1 mo): alive; clinical, laboratory and radiological response |
| **Mel et al.(16),**  **2015** | 21 yrs, female | B-symptoms with hemorrhage, jaundice and abdominal distension  PB: anemia and thrombocytopenia, no lymphocytosis initially | HSM, ascites | splenectomy: lymphoid proliferation without evidence of malignancy;  liver biopsy 8 months following first presentation:  CD3^+^ lymphocytic infiltrate suggesting T-LGL;  simultaneously to liver biopsy BM examination including banding karyotyping: T-LGL, 47XY, +8; *STAT3* negative | 1^st^ line: splenectomy and cortison pulse therapy, no long-term response;  2^nd^ line: CHOP x 6 followed by progress;  last FU (14 mo): alive, refractory course, palliative treatment |
| **Malamut et al.(18),**  **2012** | 1^st^ case: 75 yrs, female | 10-year history of celiac disease followed by its refractory course  PB: anemia  PB/BM: 39% and 25% of lymphocytes with T-LGL features based on microscopy, FACS and TCR analysis, respectively; | endoscopy: small intestine lesions | intestine biopsy: intraepithelial lymphocytes infiltration with immunophenotype and TCR rearrangement in accordance to T-LGL population found in PB and BM | 1^st^ line: Cy/cortison followed by Cy monotherapy with SD only;  2^nd^ line: ongoing MTX and budesonide;  last FU (10 years): alive; asymptomatic |
|  | 2^nd^ case: 48 yrs, female | 10-year history of celiac disease followed by its refractory course  PB: anemia, neutropenia, lymphopenia (0.200/µl)  PB: 35% of lymphocytes with T-LGL features based on microscopy, FACS and TCR analysis, respectively | endoscopy: small intestine lesions | intestine biopsy: intraepithelial lymphocytes infiltration with immunophenotype and TCR rearrangement in accordance to T-LGL population found in PB  liver biopsy: steatosis with LGLs in hepatic sinusoids | 1^st^ line: cortisone followed by ongoing CyA;  last FU (11 mo): alive; recovery of intestinal mucosa |
| **Lamy et al.(20),**  **2000** | 1^st^ case:  59 yrs, male | dyspnea, cough, B-symptoms, recurrent infections  PB: WBC 9.800/µl, T-LGL 8.100/µl, neutrophiles 0.200/µl  BM: 50% infiltration by T-LGL | CT lung: nodular infiltrates;  splenomegaly | lung biopsy: interstitial infiltration of CD3+/CD8+/CD57+ lymphocytes | 1^st^ line: MTX/cortisone followed by MTX mono;  last FU (2 mo): alive; complete resolution of lung infiltrates and cytopenia |
|  | 2^nd^ case:  19 yrs, male | dyspnea, B-symptoms  PB: WBC 3.000/µl, T-LGL 1.500/µl, neutrophiles 0.200/µl  BM: 40% infiltration by T-LGL | CT lung: normal;  HSM | lung biopsy: interstitial infiltration of CD3+/CD8+/CD57+ lymphocytes;  liver biopsy: atypical lymphocytic inltration of hepatic sinusoids;  splenectomy: red pulp infiltration with CD3+/CD8+/CD16+ lymphocytes | 1^st^ line: MTX/cortisone followed by MTX mono  last FU (2 mo): alive; resolution of dyspnea, hepatomegaly, abnormal liver tests and neutropenia |
|  | 3^rd^ case:  32 yrs, female | dyspnea, tachycardia  PB: WBC 9.600/µl, T-LGL 6.200/µl, neutrophiles 1.000/µl  BM: 40% infiltration by T-LGL | CT lung: normal | lung biopsy: Interstitial infiltration of CD3+/CD8+/CD57+ lymphocytes | 3^rd^ line*: MTX/cortisone  last FU (2 mo): alive; complete resolution of lung infiltrates and cytopenia |
|  | 4^th^ case:  28 yrs, male | dyspnea, syncope  PB: WBC 7.600/µl, T-LGL 4.600/µl, neutrophiles 0.700/µl  BM: 20% infiltration by T-LGL | CT lung: signs of pulmonary hypertension;  splenomegaly | liver biopsy: atypical lymphocytic inltration of hepatic sinusoids | 1^st^ line: cladribine x 6  last FU (NA): resolution of splenomegaly |
